# Supplementary material for: Mortality, loss to follow-up and advanced HIV disease following virologic success in West African HIV-2 patients
Source: PLoS One. 2025 Apr 2;20(4):e0317223. doi: 10.1371/journal.pone.0317223 (PMC11964237; doi:10.1371/journal.pone.0317223)
Supplement: Attachment — Submitted filename: Response to reviewers.docx [file pone.0317223.s003.docx]

**Mortality, loss to follow-up and advanced HIV disease following virologic success in West African HIV-2 patients**

**Journal requirements:**

1. When submitting your revision, we need you to address these additional requirements.

**Authors:** We would like to thank the editor for his comments. We have taken the additional requirements into account in the new version of the revised manuscript.

2. We noticed you have some minor occurrence of overlapping text with the following previous publication(s), which needs to be addressed: <https://doi.org/10.1371/journal.pone.0236642>

In your revision ensure you cite all your sources (including your own works), and quote or rephrase any duplicated text outside the methods section. Further consideration is dependent on these concerns being addressed.

**Authors:** We thank the editor for this comment, we reviewed the manuscript and removed all the overlaps with the above-mentioned paper.

3. Please provide a complete Data Availability Statement in the submission form, ensuring you include all necessary access information or a reason for why you are unable to make your data freely accessible. If your research concerns only data provided within your submission, please write "All data are in the manuscript and/or supporting information files" as your Data Availability Statement.

**Authors:** We thank the reviewer for this comment, we provided a complete statement about data availability, we were able to make the data available on a repository and provided the link: <https://figshare.com/s/a8b9fa7d5ba8a5968235>

4. One of the noted authors is a group [the EDIIMark-2 study group]. In addition to naming the author group, please list the individual authors and affiliations within this group in the acknowledgments section of your manuscript. Please also indicate clearly a lead author for this group along with a contact email address.

**Authors:** We thank the reviewer for this comment, the Study group has been removed from the author list, since the list is very long and we could not join all the members to sign the related consent as per our organization policy.

**Review Comments to the Author:**

Reviewer #1: This is a single-center observational study on HIV-2 in West Africa. While the disease is important, the information presented is not novel although as the authors mentioned, that this study joins the few studies to describe long-term outcomes (~5-¬¬6 years) among PLHIV-2. For e.g. it is not surprising that the patients remaining in care are more likely to have better outcomes with higher CD4 and a higher proportion in WHO clinical stage 1-2/A-B. Nevertheless, this study adds on to the sparse literature available. The manuscript will benefit with further English language editing if publishing in English.

**Authors:** We thank the reviewer for this comment, we reviewed the manuscript to improve English and editing.

Comments:
**Abstract / Methods**

1• It would be better to state that comparison categories included lost to follow up and dead. The current abstract appears to define loss to follow up = absent for more than 90 days and dead which is confusing.

**Authors:** We would like to thank the reviewer for this comment. We clarified this confusion in the revised manuscript as follow: “Outcomes were described as follow: in care (known alive and present during the last ART follow up visit), loss to follow-up (absent for more than 90 days and not reported dead), and dead (reported dead with a date of event)".

**Main manuscript / Under introduction**

2• Awkward language: “In addition, the 2019 WHO guidelines recommended Dolutegravir (DTG) in combination with a nucleoside reverse-transcriptase inhibitor (NRTI) backbone as the preferred first-line regimen for people initiating antiretroviral treatment (ART), thus without any difference between those living with HIV-1 and HIV-2 [14].” The reference to HIV-1 here is not required as well, as the authors are not comparing the treatment outcomes across HIV-1 and HIV-2.

**Authors:** We thank the reviewer for this comment. We agree that there was no comparison between HIV-1 and HIV-2 planned in this analysis. Therefore, we removed the phrase “thus without any difference between those living with HIV-1 and HIV-2” in the revised version of the manuscript.

**Main manuscript / Under outcomes and variables**

3•While it would be fair for the authors to collapse mortality cases with lost to follow up if the numbers are small, I am concerned about the scientific basis stated that loss to follow up is a proxy of death.

**Authors:** We thank the reviewer for this comment. We decided to combine LTFU and death because there are papers in the literature that suggest that lost to follow-up is a proxy of death ("Loss to Follow-up: A Major Challenge to Successful Implementation of Prevention of Mother-to-Child Transmission of HIV-1 Programs in Sub-Saharan Africa). We included this paper as reference in the revised manuscript.

4• The preceding sentence mentioned that the authors categorized the participants into “3 modalities” and the next they collapsed 2 of them into 1. Modalities would not be the correct word to use here, but rather categories.

**Authors**: We thank the reviewer for this suggestion that has been taken into consideration by replacing “modalities” by “categories” in the revised version of the manuscript.

5• It is not necessary to comment on the creation of a new variable. It would suffice to say that participants were categorized into 2 or 3 categories based on whether they are still on follow up or not.

**Authors:** We thank the reviewer for this comment. We adjusted the sentence accordingly in the revised version of the manuscript.

**Main manuscript / Data collection**

6• Awkward word use: Instead of abstraction, extraction would be a better word to use e.g. “A standardized questionnaire allowed extraction of data…”

**Authors**: We thank the reviewer for this suggestion, we adjusted the text accordingly in the revised version of the manuscript.

**Main manuscript / Results**

7• Was there any documented reason for the 13 HIV-2 clients to not participate in the survey? It would be interesting if yes, and this could be detailed.

**Authors:** We thank the reviewer for this comment. Among this 13 people living with HIV-2 who did not participate to the cohort, height did not consent to participate and the remaining 5 have been transferred to facilities out of the town and not part of the study sites, making them not eligible.

8• The median follow-up period for the last visit for the LTFU and dead group should be stated.

**Authors:** We thank the reviewer for this comment. The median follow-up period for the last visit for the LTFU and dead group is (40 [22 - 63] months) and can be found in the results section of the manuscript, at the third line of paragraph 2.

9• The time point of HIV-2 viral load check should be mentioned and what the median and IQR are for those who are not considered controlled.

Authors: We thank the reviewer for giving an opportunity to update and improve the quality of our manuscript. In Cote d’Ivoire, HIV-2 Viral load was not routinely prescribed like CD4 count, because it was not supported by the HIV program. Thus, HIV-2 Viral load reported in our study was sponsored by the research project and done only at two-time point in 2012 and in 2018.

We recognized that median and IQR of viral load could be more informative. However, the lab results were not always given with the number of copies making it difficult to estimate the median number of copies for all the participants. We therefore made the choice to present the conclusion of the lab set as detectable or undetectable.

**Main manuscript / Discussion**

10• The discussion does not really discuss about the findings of the research. For e.g. the research findings do not talk about opportunistic infections, but the discussion went into a paragraph of what would be helpful with opportunistic infections. In a cohort study wherein participants were initially all well-controlled, retainment in care would be more relevant rather than OI treatment.

**Authors:** We thank the reviewer for this comment. We apologize for the confusion brought in the discussion when mentioning opportunistic infection. Our study did not focus on opportunistic infections as the reviewer rightly said, but rather explored attrition (LTFU and Death) and advanced HIV disease.

In the first graph we comment on the high attrition rate, giving the causes of this high rate and extrapolate on potential strategies to improve retention to care. In the second paragraph, we comment on the high rate of advanced HIV disease, and extrapolate on solutions that increase survival of patients. we adjusted the discussion section and tried to clarify that.

11• The finding that all participants are on boosted PI/NRTI regimen could have also been commented on or discussed. Depending on the HIV-2 viral load on those who are not controlled, there is a consideration wherein II based regimens would be more helpful.

Authors: We thank the reviewer for this comment. We added a comment in the discussion section reflecting the situation of participants receiving the recommended ART regiment for HIV-2 and experiencing an uncontrolled Viral load. Indeed, these patients should be switched on II. It is true that by 2018 the II were recommended only for the third lines, but with the roll out of DTG in 2019, all these patients were systematically switched to a DTG-based regimen.

Reviewer #2:

1• Page 4, the spelling of house should be corrected. wrong "hose"; correct "house"

**Authors:** We would like to thank the reviewer for spotting this typing error. We corrected this misspelling in the update version of the manuscript.

2• On Data collection section, a standard data extraction form should have been used not a standardised questionnaire. Correct accordingly.

**Authors:** We thank the reviewer for this comment. The correct term is data extraction sheet. We have made a correction in the revised version of the manuscript.

3• Page 6, third paragraph and sentences 3 and 4 are confusing. I suggest you delete the 3rd sentence

**Authors:** We thank the reviewer for this comment. In the third sentence, we would like to show the proportions of the latest viral loads in our entire study population. However, we agree with the reviewer that it could be confused with the sentence that follows. In this revised version, we deleted this sentence as suggested by the reviewer.

4• Page 7, Table 1. Variables Age (median, IQR) years and Duration of ART (years) have 3 entries for only 2 categories. This needs to be reviewed.

**Authors:** We thank the reviewer for this valuable comment. The medians and categories of these variables overlapped and created confusion. We have distinguished them better in the new revised version of the manuscript.

5• Question: It was mentioned that all the PLHIV-2 at enrolment were of PI. During the follow-up period following the WHO recommendation to transition all patients to DTG, was this transition done? If yes, when and do you think it could affect the survival of PLHIV-2 or rate progress to advanced HIV disease? Consider addressing these elements in your work. Thanks

**Authors**: The WHO recommendation was implemented on a large scale in Côte d'Ivoire in 2019, after this study was carried out. Since then, all HIV-2 patients on NRTI/PI have been switched to dolutegravir. This transition has improved the management of PLWH-2 in virological failure by offering an alternative second-line treatment that was previously limited. We added a paragraph addressing the DTG transition in the discussion.
