# Supplementary material for: Mortality, loss to follow-up and advanced HIV disease following virologic success in West African HIV-2 patients
Source: PLoS One. 2025 Apr 2;20(4):e0317223. doi: 10.1371/journal.pone.0317223 (PMC11964237; doi:10.1371/journal.pone.0317223)
Supplement: Attachment — Submitted filename: Academic Edit comm_HIV2 West Africa.docx [file pone.0317223.s002.docx]

**Mortality, loss to follow-up and advanced HIV disease following virologic success in West African HIV-2 patients**

***General comments/suggestions***

This is an important topic to address as HIV2 is peculiar in West African countries as compared to HIV1. The authors have tried to analyze the data in the standard way and the interpretation also sounds good.

However, there is a major need to review the way the article is written-it could be benefited from editorial of English language. The authors can still expand the discussion and clearly explain to the reader the why part of similar or high LTFU/death/advanced HIV diseases among the HIV2 patients as the expectation is HIV2 is ‘less ‘virulent’ than HIV1. It could have been also possible if the context-based comparison was made between the two countries from where data was obtained.

Otherwise, the authors have tried to respond to the enquires from the peer reviewers. Hope the remaining comments/suggestions are quite constructive to further enrich this important manuscript.

***Specific commonest/suggestions***

You could have line number the article that eases the review process.

**Abstract**

*“’This emphasizes the need to improve holistic care for HIV-2 patients.”* The term holistic care is quite comprehensive for recommendation. Could the authors specify discrete recommendations that need to be done to reduce the advanced HIV diseases?

**Introduction**

The last paragraph should make sure that there is no published article in this regard- scarcity of evidence should strongly and boldly be described.

Examples:

*Raugi DN, Ba S, Cisse O, Diallo K, Tamba IT, Ndour C, Badiane NM, Fortes L, Diallo MB, Faye D, Smith RA. Long-term experience and outcomes of programmatic antiretroviral therapy for human immunodeficiency virus type 2 infection in Senegal, West Africa. Clinical Infectious Diseases. 2021 Feb 1;72(3):369-78.*

Also check why the term mortality started with capital letter here*,” … Mortality, loss to follow-up (LTFU), advanced HIV disease…’’* Please check similar issues throughout the document.

**Methods**

The following sentence is too long, and it is not clear. Please revise. Do the same throughout the document.

*“A cohort of people living with HIV-2, identified as mono-infected and followed-up in HIV clinics participating in the International Epidemiological Database to Evaluate AIDS (IeDEA) West Africa, was initiated in January 2012.”*

**Follow up**

Mix of tenses used, future and past. You may revise*,”* *In case of missed appointment, a phone-base tracking procedure* ***will take p****lace to bring the patient back to care, and if not successful, home visits can be organized for the patients who agreed and provided localization of their house at enrolment. Blood sample w****as*** *collected every six months from each patient to perform CD4 count measurements. For the purpose of this analysis, all the participants* ***were****…”*

This is a vague sentence as well, *“For the purpose of this analysis, all the participants were seen from October to December 2018 during their last appointment by the same clinician who conducted clinical examination and updated the disease clinical stage before collecting blood sample for viral load testing”*

**Data collections**

Again, mix of tenses*,”* *A structured questionnaire* ***allows*** *extraction of data from patient’s medical files, and additional data* ***were*** *obtained during face-to-face interview with patients or* ***abstracted*** *in the facility databased.”* Also check whether the word *‘abstracted****’*** is the right term to use here.

Check if the heading is “Outcomes and variables” or “Outcomes variables”. It seems only the outcome variables were operationalized. I strongly suggest to re-visit the PLOS ONE guidelines (<https://journals.plos.org/plosone/s/submission-guidelines.#:~:text=The%20PLOS%20ONE%20article%20component%20must%20comply%20with%20the%20general>)

Check why ‘Characteristic” started with capital letter here;*” Table 1: Follow-up Characteristics of ART-experienced people living with HIV-2 five years following virologic success in West Africa (N=95)”*

Gender parity-Table 1: how was the proportion of male and female became equal? Was it intentional? Please justify or check the data.

Now we are in 2024, and why could the authors not extend beyond 2018? Don’t you think the data is outdated?

Is there any difference in terms of the outcome variables between the two countries? Is it possible to include this in the result and discuss the difference?

It is expected to have a better outcome from treating HIV2 as compared to HIV1. Why was the advanced HIV diseases, LTFU and death all same or more than those patients infected with HIV1? Please try to justifying this issue further.

In conclusion ( abstract and at end of the discussion) please try to suggest specified interventions for the alarmingly high LTFU/death/Advanced HIV diseases:-Management of comorbidities, addressing adherence issue, considering a better regimens etc…

**Discussion**

It is shallow and may be expanded a bit.

Potential point of discussion could be the difference in the outcomes between the two countries (Cot’d vory and Burkin faso) or outcome similarity between HIV1 and HIV2.

Please try to trim/consolidate the limitation.
