# Supplementary material for: Mortality, loss to follow-up and advanced HIV disease following virologic success in West African HIV-2 patients
Source: PLoS One. 2025 Apr 2;20(4):e0317223. doi: 10.1371/journal.pone.0317223 (PMC11964237; doi:10.1371/journal.pone.0317223)
Supplement: Attachment — Submitted filename: Response_to_Reviewers_auresp_2.docx [file pone.0317223.s005.docx]

**Mortality, loss to follow-up and advanced HIV disease following virologic success in West African HIV-2 patients**

***General comments/suggestions***

This is an important topic to address as HIV2 is peculiar in West African countries as compared to HIV1. The authors have tried to analyze the data in the standard way and the interpretation also sounds good.

However, there is a major need to review the way the article is written-it could be benefited from editorial of English language. The authors can still expand the discussion and clearly explain to the reader the why part of similar or high LTFU/death/advanced HIV diseases among the HIV2 patients as the expectation is HIV2 is ‘less ‘virulent’ than HIV1. It could have been also possible if the context-based comparison was made between the two countries from where data was obtained.

Otherwise, the authors have tried to respond to the enquires from the peer reviewers. Hope the remaining comments/suggestions are quite constructive to further enrich this important manuscript.

***Specific commonest/suggestions***

You could have line number the article that eases the review process.

**Abstract**

“’This emphasizes the need to improve holistic care for HIV-2 patients.” The term holistic care is quite comprehensive for recommendation. Could the authors specify discrete recommendations that need to be done to reduce the advanced HIV diseases?

Authors: We would like to thank the reviewer for this comment. We agree that “holistic care” is definitely quite comprehensive to be part of a conclusion. We have updated the abstract by rewording it and adding practical take home messages. The conclusion has been updated accordingly by summarizing the key messages regarding to “holistic” care, discussed in the discussion section. Page 2, lines 52-54.

**Introduction**

The last paragraph should make sure that there is no published article in this regard- scarcity of evidence should strongly and boldly be described.

Examples:

*Raugi DN, Ba S, Cisse O, Diallo K, Tamba IT, Ndour C, Badiane NM, Fortes L, Diallo MB, Faye D, Smith RA. Long-term experience and outcomes of programmatic antiretroviral therapy for human immunodeficiency virus type 2 infection in Senegal, West Africa. Clinical Infectious Diseases. 2021 Feb 1;72(3):369-78.*

Also check why the term mortality started with capital letter here*,” … Mortality, loss to follow-up (LTFU), advanced HIV disease…’’* Please check similar issues throughout the document.

Authors: We thank the reviewer for this comment. We have corrected this capitalization in the revised version of the manuscript. Page 3, line 81.

**Methods**

The following sentence is too long, and it is not clear. Please revise. Do the same throughout the document.

*“A cohort of people living with HIV-2, identified as mono-infected and followed-up in HIV clinics participating in the International Epidemiological Database to Evaluate AIDS (IeDEA) West Africa, was initiated in January 2012.”*

Authors: We thank the reviewer for this comment. In the revised version of the manuscript, we have split this long sentence into two sentences to make it easier to understand. Page 4, lines 85-87.

**Follow up**

Mix of tenses used, future and past. You may revise*,”* *In case of missed appointment, a phone-base tracking procedure* ***will take p****lace to bring the patient back to care, and if not successful, home visits can be organized for the patients who agreed and provided localization of their house at enrolment. Blood sample w****as*** *collected every six months from each patient to perform CD4 count measurements. For the purpose of this analysis, all the participants* ***were****…”*

Authors: We thank the reviewer for this comment. We have standardized the (past) tense in the revised version of the manuscript. Page 4, line 96.

This is a vague sentence as well, *“For the purpose of this analysis, all the participants were seen from October to December 2018 during their last appointment by the same clinician who conducted clinical examination and updated the disease clinical stage before collecting blood sample for viral load testing”*

Authors: We recognize that this sentence is not very precise. We have reworded it in the revised manuscript to make it more understandable. Page 4, lines 99-103.

**Data collections**

Again, mix of tenses*,”* *A structured questionnaire* ***allows*** *extraction of data from patient’s medical files, and additional data* ***were*** *obtained during face-to-face interview with patients or* ***abstracted*** *in the facility databased.”* Also check whether the word *‘abstracted****’*** is the right term to use here.

Authors: We've changed the sentence by harmonizing the tenses. The reviewer will find these changes in the revised version of the manuscript. Page 4, lines 108-110.

Check if the heading is “Outcomes and variables” or “Outcomes variables”. It seems only the outcome variables were operationalized. I strongly suggest to re-visit the PLOS ONE guidelines (<https://journals.plos.org/plosone/s/submission-guidelines.#:~:text=The%20PLOS%20ONE%20article%20component%20must%20comply%20with%20the%20general>)

Authors: The appropriate topic is 'Outcome variables', not 'Outcomes and variables'. We have made a correction in the revised version of the manuscript. Page 5, line 116.

Check why ‘Characteristic” started with capital letter here;*” Table 1: Follow-up Characteristics of ART-experienced people living with HIV-2 five years following virologic success in West Africa (N=95)”*

Authors: We have removed this major feature in the revised version of the manuscript. Page 7, line 172.

Gender parity-Table 1: how was the proportion of male and female became equal? Was it intentional? Please justify or check the data.

Authors: All patients with an undetectable viral load in 2012 were included in this study, regardless of gender. The equal proportion of men and women included in the analysis was by sheer coincidence. This result has already caught our attention during the data analysis phase, and even during the review by co-authors. After a second review, we confirmed it.

Now we are in 2024, and why could the authors not extend beyond 2018? Don’t you think the data is outdated?

Authors: The reviewer points out an interesting issue about this paper. First, after the success of the DLT transition into most of HIV programs, learning through knowledge building and sharing is still needed. Even our data seems outdated exclusively based on the data collection period, we think that they should contribute to more learning about the improving of PLHIV care and could serve programs managers as the outcomes of their efforts and progresses. In addition, as documenting in the limitation paragraph, at the discussion section, lessons learnt through this study suggest the potential benefic effect of Protease inhibitors (PI) - Dolutegravir transition on improving the management of PLWH-2 in virological failure by offering an alternative second-line treatment.

Based on these reasons, we would like to submit again the manuscript and call for its publication to nourish the science and contribute to the experience learned on the long-term outcomes of PLHIV under PI-based ART.

Is there any difference in terms of the outcome variables between the two countries? Is it possible to include this in the result and discuss the difference?

Authors: We thank the reviewer for this comment. For logistical reasons, the PLHIV-2 from Burkina Faso included in the 2012 cohort did not contribute to this analysis. This study only included the Côte d'Ivoire cohort. We have added this specificity to the method section to make it clearer. The revised version of the manuscript was also updated. Page 4, line 89.

It is expected to have a better outcome from treating HIV2 as compared to HIV1. Why was the advanced HIV diseases, LTFU and death all same or more than those patients infected with HIV1? Please try to justifying this issue further.

Authors: These similar or even higher proportions in PLWH-2 could be explained by the fact that prior to the recommendation of integrase inhibitors based ART, the therapeutic options for PLWH-2 were very limited. The PI-based ART regimen were indicated for first-line regimen, so ARVs resistances to this first-line made challenging the PLHIV-2 management. This situation exposed these patients to a higher risk of death if first-line ART failed. In addition, as with HIV-1, PLWH-2 presented at a late stage with severe immunosuppression, most commonly associated with opportunistic infections. In the revised version of the manuscript, we have modified the discussion to include this rationale. Page 9, lines 206-210.

In conclusion (abstract and at end of the discussion) please try to suggest specified interventions for the alarmingly high LTFU/death/Advanced HIV diseases:

-Management of comorbidities, counseling for adherence and retention, addressing adherence issue, considering a better regimens etc…

Authors: We thank the reviewer for this comment. Strategies to improve the management of PLWH-2 and to prevent advanced HIV disease according to WHO recommendations were expanded in the discussion. We have summarized some strategies in the revised version of the manuscript (including abstract). Page 10, lines 224-229.

**Discussion**

It is shallow and may be expanded a bit.

Potential point of discussion could be the difference in the outcomes between the two countries (Cote d’Ivoire and Burkin faso) or outcome similarity between HIV1 and HIV2.

Please try to trim/consolidate the limitation.

Authors: The data collected in this study only covered HIV clinics in Côte d'Ivoire, as reported earlier in the method section. We were therefore unable to make a comparison with data from Burkina Faso, which was not collected during the 2018 monitoring visit.

We have reduced the limitations section as suggested by the reviewer.
