# Supplementary material for: Mortality, loss to follow-up and advanced HIV disease following virologic success in West African HIV-2 patients
Source: PLoS One. 2025 Apr 2;20(4):e0317223. doi: 10.1371/journal.pone.0317223 (PMC11964237; doi:10.1371/journal.pone.0317223)
Supplement: Attachment — Submitted filename: Final comments_ZGD_Ac edit_Dec 23_2024.docx [file pone.0317223.s004.docx]

***Final comments to be fixed before consideration for publication***

**Abstract**

Replace DTG with dolutegravir. Please look again in the manuscript—all abbreviations should be defined during their first instance if they are to be used again.

**Introduction**

“Data from the ANRS CO5 HIV-2 Cohort Study”. Same comment as above-- what is ANRS CO5 in, “ Data from the **ANRS CO5** HIV-2 **C**ohort **S**tudy”? Also why capital letters?

**Methods**

‘’A cohort of people living with HIV-2 (PLHIV-2)…’’ only PLHIV-2 is enough. You have already defined.

Check again if it is important to start these phrases with capital letters, “ As per the **N**ational **G**uidelines for PLHIV, clinically…”

Check if the following long sentence could be revised to two sentences.

’’ Based on their follow up status, participants were categorized as in care (still in the follow-up and receiving ART, including being transferred out), dead and LTFU, and the last two were subsequently

combined in one category in the analyses as they both constitute a negative outcome and it is always challenging to know among the LTFU the proportion of death (19).”

***May be replaced with;***

‘’ Based on their follow up status, participants were categorized as in care (still in the follow-up and receiving ART, including being transferred out), dead and **LTFU. The last** two were subsequently

combined in one category in the analyses as they both constitute a negative outcome, and it is

always challenging to **the proportion of death** know among the LTFU (19).’’

Replace “Chi2-square or Fisher’s exact tests ..” with “Chi-square (X2) or Fisher’s exact tests ….”

**Replace** “Prior to the enrolment in the cohort, each participant received detailed information about the study and signed an informed consent form.”

**With**

“Prior to the enrolment in the cohort, detailed information about the study was given to each study participants and a signed informed consent was obtained .”

What is “PACCI”? Please define.

Check if “PI based ART regimen…” Could be written as, “ Protease inhibitors (PI) based ART regimen…”

Check if the sentence, “At the last follow-up visit, the median CD4 count was 518 [353 – 639] cells/mm3 not different in those remaining in care compared to those dead/LTFU (534 Vs 431; p=0.09).”

**could better be written as**,

“At the last follow-up visit, the median CD4 count was 518 [353 – 639] cells/mm3, and there is no statistically significant difference in those remaining in care compared to those dead/LTFU (534 Vs 431; p=0.09).”

**Discussion**

“Moreover, since 2019, national HIV treatment guidelines aligned on WHO guidelines

recommended Tenofovir plus Lamivudine plus dolutegravir (TLD) regimen as first-line and the

transition of all ART-experienced clients, while the use of boosted protease inhibitors (PI/r) as

second-line.” This is not easy to understand. Please simplify or written in a simple sentence for easy understanding.

**Conclusions**

Which one is right?

“..**five years** after an initial virologic success…” in the abstract conclusion

**or**

“Our study indicates that **six years** after being reported stable,…” in the final conclusion?
